# Biodegradable polymeric occluder with controllable locking structure for closure of atrial septal defect via interventional treatment

**DOI:** 10.1093/rb/rbaf016

**Published:** 2025-03-20

**Authors:** Daokun Shi, Yahong Kang, Weijie Wang, Ruili Liu, Quansheng Tang, Zhaomin Li, Hongyan Jiang, Jiandong Ding

**Affiliations:** State Key Laboratory of Molecular Engineering of Polymers, Department of Macromolecular Science, Fudan University, Shanghai 200438, China; State Key Laboratory of Molecular Engineering of Polymers, Department of Macromolecular Science, Fudan University, Shanghai 200438, China; Shanghai Key Laboratory of Interventional Medical Devices and Equipment, Shanghai MicroPort Medical Group Co, Ltd, Shanghai 201203, China; AccuPath Group Co., Ltd, Jiaxing 314000, China; Shanghai Key Laboratory of Interventional Medical Devices and Equipment, Shanghai MicroPort Medical Group Co, Ltd, Shanghai 201203, China; AccuPath Group Co., Ltd, Jiaxing 314000, China; State Key Laboratory of Molecular Engineering of Polymers, Department of Macromolecular Science, Fudan University, Shanghai 200438, China; Shanghai Key Laboratory of Interventional Medical Devices and Equipment, Shanghai MicroPort Medical Group Co, Ltd, Shanghai 201203, China; AccuPath Group Co., Ltd, Jiaxing 314000, China; Shanghai Key Laboratory of Interventional Medical Devices and Equipment, Shanghai MicroPort Medical Group Co, Ltd, Shanghai 201203, China; AccuPath Group Co., Ltd, Jiaxing 314000, China; Shanghai Key Laboratory of Interventional Medical Devices and Equipment, Shanghai MicroPort Medical Group Co, Ltd, Shanghai 201203, China; State Key Laboratory of Molecular Engineering of Polymers, Department of Macromolecular Science, Fudan University, Shanghai 200438, China

**Keywords:** atrial septal defect, interventional treatment, cardiac occluder, biodegradable polymer, poly(L-lactide)

## Abstract

Atrial septal defect (ASD) is one of the major congenital heart diseases, and transcatheter closure with a cardiac occluder is a modern method to treat ASD with the advantage of mini-invasiveness over traditional surgical closure. While current occlusion devices are mainly made of non-degradable nitinol with superelasticity, the permanent existence of a metal *in vivo* may trigger potential complications and especially has an adverse effect on the heart development for children. However, it is challenging to invent a superelasticity-free occluder that can be delivered through a catheter but firmly locked after being opened at the target site; it is also much desired for research and development to quickly assess the feasibility of a superelasticity-free occluder *in vitro*. Herein, a biodegradable poly(L-lactide) (PLLA) occluder composed of a braided PLLA frame as the skeleton and a nonwoven PLLA fabric as the flow-blocking membrane is developed, and a controllable locking structure is designed to enable firm closure for a device even without superelasticity. We also suggest and justify a series of *in vitro* methods to assess the efficacy of the biodegradable occluder, and the results confirm the reliability of locking, water-blocking, mechanical strength and degradability. It is found that the PLLA fabric with moderate fiber density is optimal for surface endothelialization. We also carry out biological assessments; significant endothelialization and alleviated inflammation response are observed after 6 months of subcutaneous implantation into rabbits. The porcine model illustrates that the biodegradable polymeric occluder can be successfully implanted into the atrial septum via transcatheter intervention; the follow-ups have confirmed the safety and efficacy of this biodegradable polymeric occluder with the controllable locking structure.

## Graphical Abstract

**Figure:**
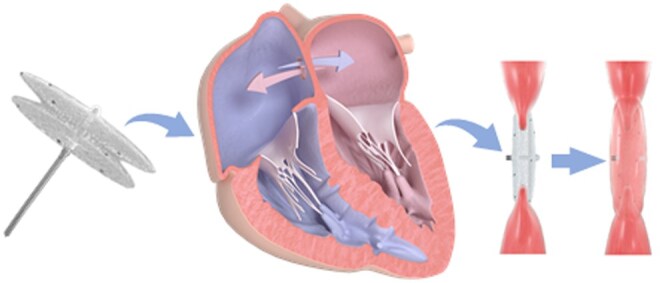

## Introduction

Atrial septal defect (ASD) is the third most prevalent congenital structural heart disease, accounting for approximately 5–10% of congenital heart diseases; statistically, one newborn suffers from this disease in every 1000 newborns [1]. ASD leads to a series of complications, including inadequate blood supply, arrhythmia, pulmonary infection, congestive heart failure and pulmonary arterial hypertension [2–6]. The traditional treatment primarily involves open-chest surgery to suture the defect, which is of high risk and postoperative sequela while leaving extensive scarring on the patient [7]. Transcatheter interventional therapy [8–11] can well achieve ASD closure [12]. Within 3–6 months after implantation of an occlusion device, gradual endothelialization and self-repair occur on the defect. Compared with the traditional surgical closure, this interventional approach has the advantages of minimal invasiveness, high surgical safety, short hospitalization time and low complication rate [13–16], making it increasingly favored as an alternative option.

A metal cardiac occluder is the mainstream interventional device utilized in clinical practice [17–21]. Notably distinguished by its exceptional superelastic performance, the Ni–Ti alloy occluder exhibits remarkable capability for recovering to its original shape after being pushed out of the delivery sheath, and outstands its blocking effect [22–24]. Nevertheless, the non-degradable nature of metal occluders results in permanent retention within body, which may trigger potential complications including friction-induced damage, cardiac erosion, inflammation, arrhythmia, metal allergy, pericardial effusion and thrombus formation. Furthermore, the presence of a metallic occluder obstructs the atrial septal channel and thus impedes further effective intervention for acquired heart diseases [25–29]. This situation also proves detrimental toward pediatric cardiac development.

The next-generation atrial septal occluder is supposed to provide a temporary scaffold for the defect to self-repair [30–32]. Once the defect is completely endothelialized, the mission of an occluder has accomplished. An ideal occlusion device should be biodegradable, and the degradation products are non-toxic and can be fully absorbed [33, 34]. A biodegradable occluder can offer a viable solution addressing issues associated with non-degradability and obstruction of the atrial septal channel that metal occluders cause, but research and development (R&D) of a biodegradable occluder faces a series of challenges. Owing to lack of superelasticity, a biodegradable occluder can hardly recover to its original state; it is also difficult for a physician to implant this occluder to the target position, which increase the risks of inadequate closure or residual shunting. Some researchers have integrated lock-in mechanisms or recoil systems into biodegradable occluders aiming at compensating the inherent lack of elasticity [30, 31]. Notable examples include those developed by LifeTech [35, 36], Lepu medical [37, 38] and Jinkui Medical [39] characterized by their internalized lock-in components, ensuring secure fixation between left-right disks. Several medical institutions in the United States have collaborated to devise a biodegradable occluder for ASD or patent foramen ovale (PFO) with a microspring embedded inside, capable of providing requisite elasticity to realize shape recovery upon catheter delivery [40]. While these approaches prove efficacious to solve the problem of insufficient resilience of a biodegradable occluder, continuous exploration remains imperative toward developing advanced structural design.

When a cardiac occluder is implanted to the target defect position, it is anticipated that the double disks of an occluder can achieve faster and more uniform endothelialization, and it is of vital significance for the self-repair of the septal defect. Research on how to facilitate a smooth endothelialization on the surface of a biodegradable occluder holds great significance. The outside of the occluder disk mainly consists of a braided mesh and a flow-blocking membrane, and the flow-blocking membrane is sewn under the braided mesh. Since the holes of the braided mesh are much larger than endothelial cells, the cells are more prone to adhere, proliferate, and migrate on the surface of the flow-blocking membrane at the bottom of the braided mesh (Figure S1). Therefore, the shape, surface hydrophilicity and composition of the flow-blocking membrane can influence the endothelialization on the occluder surface. Coating CD34 antibody on a biodegradable flow-blocking membrane can promote the endothelialization of a biodegradable occluder [41]; this application in medical devices belongs to a combination of drug and medical devices and will significantly increase the difficulty and cost of preclinical and clinical evaluations. Effective promotion of endothelialization via the microstructure of the flow-blocking membrane has higher practicability [42].

Whether a medical device is safe and effective depends much on preclinical reliable evaluation methods. To access a metal cardiac occluder is available from the standards of ISO 22679:2021 and YY/T 1553-2017. Nevertheless, there is no relevant evaluation standard for a biodegradable occluder. How to effectively and comprehensively evaluate the performances of a biodegradable occluder is worthy of exploration.

**Figure 1.**
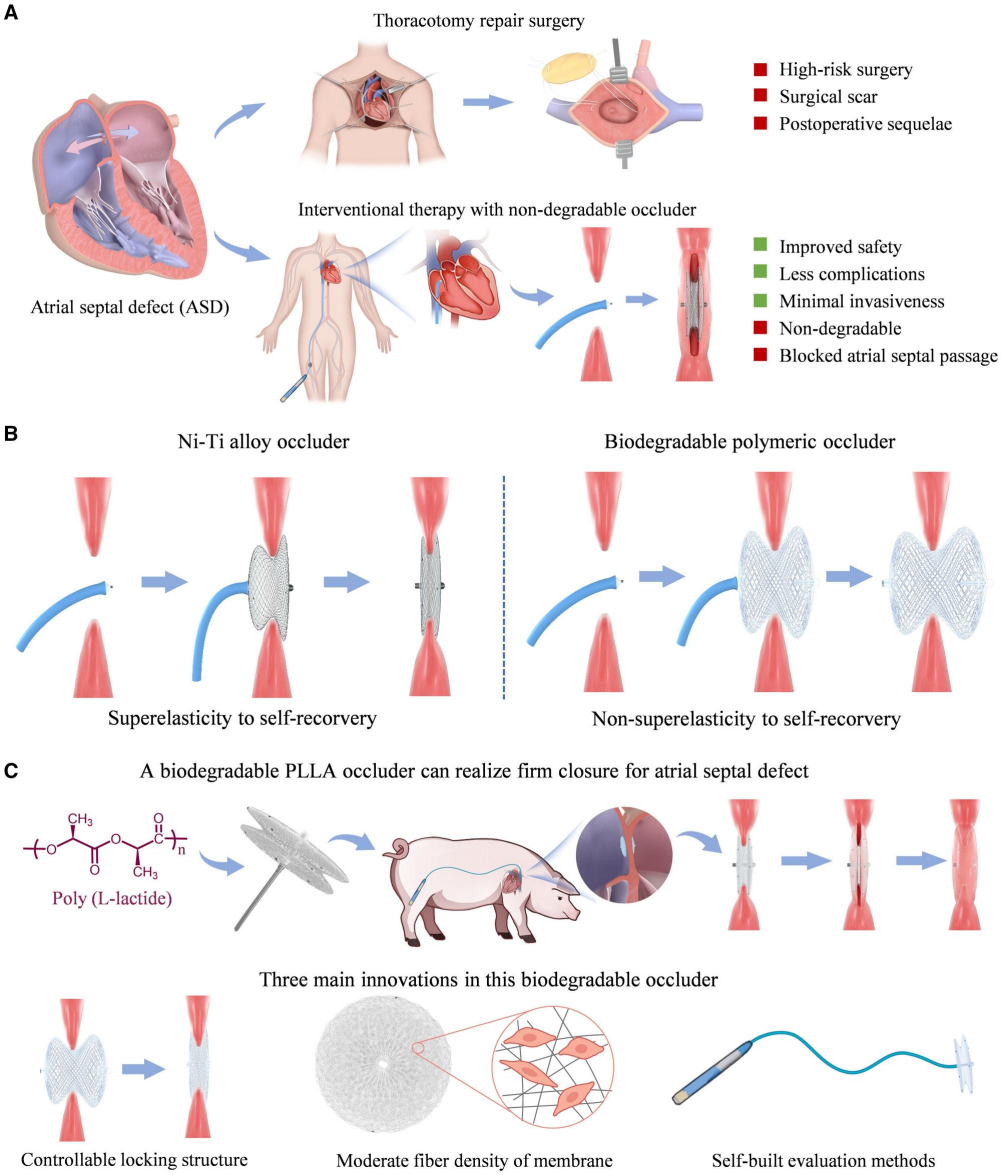
Motivation and the basic idea to develop a biodegradable occluder with controllable locking structure. (**A**) Atrial septal defect (ASD) is mainly treated with thoracotomy repair surgery and interventional therapy with a nondegradable occluder in clinical practice, and a series of issues are required to be improved. (**B**) Transcatheter closure with nickel-titanium (Ni–Ti) alloy occluder and biodegradable polymeric occluder for ASD closure. While a Ni-Ti alloy occluder can, owning to its superelasticity, easily recover to the initial shape after being pushed out from delivery sheath, a biodegradable occluder can hardly recover to the initial shape. (**C**) R&D of a biodegradable PLLA occluder and the corresponding three main innovation issues: (i) Controllable locking structure to realize the “superelasticity” function; (ii) moderate fiber density of membrane to promote endothelialization; (iii) self-built evaluation methods to improve the effectiveness and reliability.

The present paper provides a biodegradable ASD occluder with a controllable locking structure easy to operate with the motivation and basic idea schematically presented in Figure 1. This occluder is composed of braided skeleton of poly(L-lactide) (PLLA) and nonwoven membrane of PLLA completely absorbed within 2–3 years. Importantly, the reversible operations of locking and unlocking can be realized by using an elastomer component installed inside with the characteristics of extrusion deformation, ensuring effective locking and rapid unlocking. Aiming to effectively evaluate the performances of this biodegradable occluder, a series of self-built methods are proposed and justified. Furthermore, the adhesion, proliferation and migration of endothelial cells are promoted, achieving faster endothelialization by regulating the fiber pore size and density of the flow-blocking membrane of occluder. The feasibility of this biodegradable occluder is demonstrated by subcutaneous implantation of rabbits and the implantation of large animals.

## Materials and methods

### Preparation of PLLA monofilament and membrane

The PLLA material was synthesized similar to our previous publication [43]. The molding process of PLLA monofilament includes extrusion of the original filament and secondary stretching orientation. First, the PLLA particle was vacuum dried at 80°C for 12 h and added into the extruder, and the single screw extrusion parameters were set, including the screw temperature and extrusion speed, the acceptance range of the monofilament diameter and the winding rate. During the extrusion process, the extrusion parameters were adjusted until the surface of monofilament was smooth and the diameter gradually stabilized, and then the original monofilament was continuously wound. The original filament was stretched in a hot water bath (80°C) and well oriented in the axial direction. The orientation tensile ratio was set by the speed ratio of the runners to regulate the diameter of PLLA monofilament, and the ratio of 1:4 was chosen to ensure the right diameter, better appearance and suitable elongation at break of PLLA monofilament. The stretched monofilament was naturally cooled at room temperature.

The PLLA nonwoven fabric was prepared by melt spinning. The PLLA material was melted using a screw extruder with the melt flow controlled by a filter and metering pump. The melt PLLA flowed through the spinneret to form fine fibers and cooled down under N_2_ flow. The fibers were drawn onto a receiving net to fabricate the nonwoven fabric. Finally, hot pressing and bonding were applied to certain areas of the fabric to avoid slippage between fibers.

A scanning electron microscope (QUANTA 200 FEG, USA) was used to observe the PLLA monofilament and nonwoven fabric under an accelerating voltage of 10 kV in the secondary electron mode.

The electronic universal testing machine (Instron 5943, USA) was used to test the mechanical properties. The tensile speed was set as 10 mm/min. Four independent specimens were detected for each group of PLLA monofilament and nonwoven fabric.

The molecular weight was determined using gel permeation chromatography (GPC) (Waters, 2695, USA) with a refractive index detector. High-performance liquid chromatography grade chloroform was used as a solvent. Analysis was performed at 35°C with a flow rate of 1 ml/min through three columns (Waters HR3, HR4, HR5) and a calibration range of 5000–300 000 Da calibrated with polystyrene standards.

X-ray diffraction (XRD) was performed with a D500 diffractometer (Siemens, Germany). CuKα radiation with wavelength 0.154 nm was used at a voltage of 40 kV and current of 25 mA. The samples were scanned following a step-scanning method with a step-size of 0.05° and 2-s interval in the range from 10° to 35° of the diffraction angle (2θ).

### Fabrication of the biodegradable PLLA occluder

The fabrication process of the biodegradable PLLA occluder is described as follows. First, the braided tubing was woven using a program-controlled braiding machine with 36-spindle PLLA monofilaments, and then the key locking components including an elastic connecting rod and a locking ring were assembled and fixed at the two ends of a fixed-length braided tubing. After that, the braided skeleton installed with the locking device was assembled into the metal molds, and the assembled molds were placed in a 120°C drying oven for heat set for 20 min, and naturally cooled at room temperature to obtain the double-disk skeleton. Two pieces of PLLA nonwoven fabrics were loaded into the inside of the double-disk skeleton, and sutured and fixed along the edge of the disk surface using biodegradable suture (MB66G, Johnson & Johnson). Finally, the developing markers were installed on the two disks, waist and the two ends. Under the same process, 30 PLLA occluders were fabricated and stored at −20°C. A digital microscope was used to observe and record the occluder at the initial state, the delivery state and locked state by stretching the both ends.

Considering the potential insufficient resilience of the biodegradable polymeric occluder [30, 31], we carried out ABAQUS finite element simulation to simulate the structure of the biodegradable occluder and analyzed whether the occluder can recover to its initial state after being stretched to the delivery state. The model establishment and calculation of the specific finite element simulation analysis were entrusted to the department of numerical simulation (Shanghai MicroPort Medical Group Co., Ltd).

### Physical performance evaluation

A universal testing machine was used to test the tensile stresses of the occluder, the connecting rod, the connecting wire rope, and the braided frame. The tensile rate was set to 10 mm/min. In addition, we recorded the tensile strain–stress of the biodegradable occluder from the locked state to the unlocked state and from the initial state of double disks to the tube-like delivery state.

We tried to employ a fixation force test to assess *in vitro* the fixing firmness of the occluder. A polydimethylsiloxane (PDMS) sheet with 10 cm × 10 cm × 4 mm of length × width × thickness was processed, and a circular hole with a diameter of 10 mm was punched in the center of the PDMS sheet. The biodegradable PLLA occluder was placed through the hole of the sheet, and the left and right disks were located on both sides of the hole, the connecting rod was pulled to lock the occluder, and the left and right disks were clamped at the hole. When the PDMS sheet was fixed, the locking force of the occluder pushed out of the hole from the left disk was tested using universal testing machine. A metal occluder (ASD occluder, KW081620, Dongguan Kewei Medical Instrument Co., Ltd) was used as the control.

We introduced two methods to evaluate the simulated water-blocking effect of an occluder. In one method, a cylinder with a diameter of 20 cm, a height of 35 cm and a bottom thickness of 4 mm was processed, and a circular hole with a diameter of 10 mm was punched at the bottom. The biodegradable PLLA occluder was locked on the hole with the left disk inward (left atrium, higher blood pressure) and the right disk outward (right atrium, lower pressure). Pure water was added to the cylinder with a height of 30 cm. The amount of water flowing out from the bottom within 5 s was tested and recorded. The water outflow without any occluder was used as the blank group. The water outflow without any occluder was as *V*_0_, and the water flow measured by installing the occluder at time *t* was recorded as *V_t_*. The blocking rate of water flow was calculated as follows:


$$\text{Blocking}\;\text{rate}\;\text{of}\;\text{water}\;\text{flow}=\left(V_{0}-V_{t}\right)/V_{0}$$

In another method, a rectangular water tank of 30 cm × 10 cm × 20 cm (length × width × height) was processed, separated by a partition in the middle, and the thickness of the partition was 4 mm, with a circular hole of 10 mm in the middle of the partition. The occluder was fixed at the circular hole, with the large disk on the right and the small disk on the left. Pure water was added to both sides of the water tank. The water level on the left was at the bottom of the occluder, and the water level on the right was 10 cm above the top of the occluder. The timing was started and the height of the liquid level on the right was continuously recorded at different times. No occluder was placed as the blank, and a metal occluder of the same size was placed as the control group.

### *In vitro* degradation evaluation

The occluder samples were immersed in phosphate buffer saline (PBS, pH 7.4 ± 0.2) and placed in a 60°C water bath to start degradation. The accelerated *in vitro* degradation lasted for 56 days, with three parallel samples at each time point (1, 3, 7, 14, 28 and 56 days). After the degradation experiment was completed, the samples were removed from PBS and rinsed with distilled water, and the samples were placed at room temperature to dry to constant weight. The appearances of the occluder samples after degradation were observed and recorded using an optical microscope. The mass loss of the samples after degradation was calculated by weighing.

Furthermore, 37°C degradation tests were carried out with the biodegradable occluder immersed in PBS and placed in a 37°C shaking bath. The degradation period was set to 1, 3 and 6 months, and PBS was replaced regularly to keep the pH value within the range of 7.4 ± 0.2. After the degradation experiment was completed, the samples were removed from PBS and rinsed with distilled water, and then the samples were placed at room temperature to dry to constant weight. An optical microscope was used to observe the occluders, the mass losses after degradation was calculated by weighing, and the locking force and flow-blocking effect of the degraded occluders were tested according to the methods mentioned above.

### *Ex vivo* simulated implantation

To effectively simulate the *ex vivo* implantation of the occluder, a pig heart was used to build septal model to carry on the delivery process. First, the left and right atria were determined, and the 10 F delivery sheath was located and passed through the defect. The biodegradable PLLA occluder was loaded into the 10 F delivery sheath, slowly pushed out the left disk, and the connecting wire rope was withdrawn to unfold the left disk. Then the delivery sheath was further withdrawn to unfold the right disk, and the connecting wire rope was pulled to lock the left and right disks.

The occluder loaded in the 10 F delivery sheath was placed under digital subtraction angiography (DSA). The left disk, waist and right disk of the biodegradable occluder were slowly pushed out, and the occluder was locked. The position changes during the process were observed and recorded under DSA.

### Biological safety evaluation of the biodegradable occluder

Chemistry pentathlon is the basic requirement for a medical device. First, the occluder sample was extracted at 37°C for 72 h at a ratio of 10 ml/piece, and the test solution was obtained after separating the sample from the liquid. The same volume of water in a glass container at 37 ± 1°C for 72 h was taken as a blank-control solution. The reducing substance test was performed according to 5.2.1 of GB/T14233.1-2008; the pH test was performed according to 5.4.1 of GB/T14233.1-2008; the evaporation residue test was performed according to 5.5 of GB/T 14233.1-2008; the heavy metal test was performed according to the method in 5.6.1 of GB/T 14233.1-2008; the ultraviolet absorbance test was performed according to 5.7 of GB/T 14233.1-2008.

Cytotoxicity and hemolysis tests were performed according to the standard method of ISO 10993-12: 2012. The biodegradable PLLA occluder sample was extracted at an extraction ratio of 0.2 g/mL.

### Cell adhesion and subcutaneous implantation in rabbit

To study the adhesion and proliferation of endothelial cells on the surface of our biodegradable occluder, nonwoven fabrics were chosen as the object to replace the whole occlusion device. Three kinds of biodegradable nonwoven fabrics with different surface densities (20, 40 and 80 g/m^2^) were prepared. They were first cut into discs with the sizes equivalent to the hole diameter of a 24-well plate and then were soaked and rinsed with pure water for 3 times and 10 min each time. After that, the PLLA discs were naturally dried and packed into an ethylene oxide sterilized bag. All preparation procedures were performed in an ISO Class 8 cleanroom. The prepared PLLA membranes were sterilized with ethylene oxide. The samples were placed in 24-well culture plates and spread flat. Considering that the PLLA membranes would float in the cell culture, all samples were pressed to the bottom of 24-well plates with sterilized polytetrafluoroethylene hollow cylinder inserts. Human umbilical vein endothelial cells were inoculated on the surface of the nonwoven fabrics at a density of 3.0 × 10^4^ cells per well. The cells were cultured in a cell culture incubator at 37°C and 5% CO_2_. After culture for 6, 24 and 72 h, the cells were rinsed with PBS and then treated with 4% paraformaldehyde for 20 min. The cells were further treated with 0.1% Triton-100 for 5 min, followed by PBS for 3 times. After that, cells were stained with Phalloidin-FITC (10 μM in 500 μl PBS) for 30 min, and washed with PBS for 3 times. Next, cells were stained with 2 μg/ml 4′,6-diamidino-2-phenylindole for 5 min and washed for 3 times. Five fields per well were randomly chosen in a fluorescence microscope, and the cell number and spreading area were counted using the software Image J.

Three New Zealand white rabbits were used as model animals for subcutaneous implantation of occluders. Rabbits were anesthetized by injection of sodium pentobarbital solution into the ear at a dose of 40 mg/kg⋅body weight, the hair in the surgical area was shaved, and the exposed skin was disinfected with 75% alcohol cotton balls. Two occluders were implanted in every rabbit (one site in the experimental group and one site in the control group). After 1, 3 and 6 months of implantation, the animals were sacrificed, the implants and surrounding tissues were cut off, fixed with 4% paraformaldehyde, embedded in paraffin, sectioned and HE stained. Three fields of view were randomly selected under each section for photography and double-blind reading, and the local histopathological reaction was evaluated semi-quantitatively.

### *In vivo* transcatheter implantation in pig and follow-ups

White pigs (about 40–50 kg) were chosen for the implantation of occluders. First, the pigs were anesthetized with sodium pentobarbital solution to ensure that the pigs were painless and unconscious. An incision was made in the pig's thigh to expose the femoral vein, and a catheter was inserted through the femoral vein. The catheter was gradually pushed into the heart, passed through the right atrium to the atrial septum, and the atrial septum was punctured with an atrial septal puncture needle. A 10 F delivery sheath was passed through the atrial septum, and the contrast agent was injected to check whether or not the atrial septum puncture and modeling were successful. Under DSA observation, the biodegradable PLLA occluder was pushed along the delivery sheath to the atrial septum, the left disk was pushed out and the connecting wire rope was withdrawn. The delivery was further withdrawn to release the right disk. The connecting wire rope was fixed, the connecting catheter was pushed forward, and the two disks were locked together. The contrast agent was injected, and the position of the atrial septal occluder was observed and confirmed under DSA. The connecting catheter and the delivery wire rope were rotated and released in turn, and the contrast agent was injected again to confirm that the occluder was securely released. The delivery wire rope and delivery sheath were withdrawn in turn, and the pig’s thigh wound was sutured and disinfected.

### Statistics

Student’s *t* tests were carried out to make comparison between two groups. The difference was regarded as significant if *P* < 0.05. We denote “*” as 0.01 ≤ *P* < 0.05, “**” as 0.001 ≤ *P* < 0.01, “***” as *P* < 0.001, and “n.s.” as no significant difference.

## Results

### Preparation and characterizations of PLLA monofilament and nonwoven fabric

**Figure 2.**
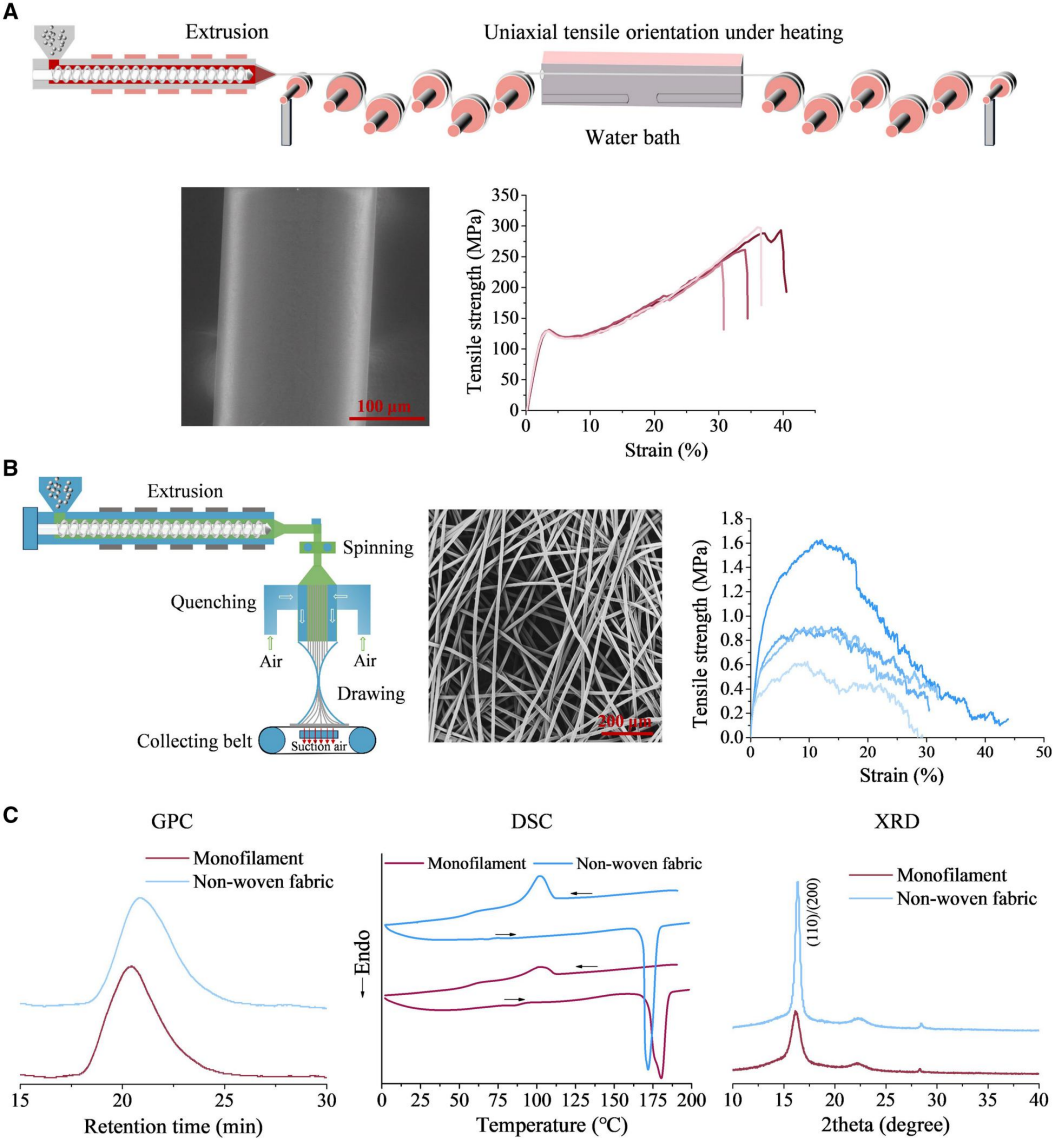
Preparation of PLLA monofilament and nonwoven fabric and their basic properties. (**A**) Schematic diagram of the processing of a PLLA monofilament including mainly extruding and uniaxial stretching under heating, and typical SEM image and stress–strain curve of the as-prepared PLLA monofilament. (**B**) Schematic diagram of the processing of a PLLA nonwoven fabric including mainly melt extruding, spinning, quenching, air drawing and collecting, and typical SEM image and stress–strain curve of as-prepared PLLA nonwoven fabric. (**C**) GPC, DSC and XRD curves of the resultant PLLA monofilament and nonwoven fabric. The arrows in DSC curves indicate the heating or cooling procedures. The numbers in XRD curves indicate the miller indexes of the facets with respect to the corresponding diffraction peaks.

Our biodegradable ASD occluder is made of PLLA monofilament and PLLA nonwoven fabric, which were, respectively, prepared with melt extrusion and melt spinning of PLLA raw materials as shown in Figure 2. The diameter of the PLLA monofilament was regulated by the speed ratio of the runners. The prepared PLLA monofilament was transparent and had a smooth surface and a diameter of about 150 μm. The stress–strain curve of the PLLA monofilament indicated a uniform and stable trend, with a tensile strength about 300 MPa and an elongation at break of more than 30%, which can meet the requirements of programmed braiding machine. The number average molecular weight *M_n_* of the PLLA monofilament read 162 kDa; the molar mass dispersity defined as weight average molecular weight *M_w_* over *M_n_* was 1.6. The XRD spectrum showed typical α-crystal peaks.

The preparation process of the PLLA nonwoven fabric included extrusion, spinning, quenching, drawing, collecting, hot pressing and bonding. The SEM image showed that the PLLA fibers of nonwoven fabric (40 g/m^2^) were evenly arranged, and the fiber diameter was between 5–10 μm. The tensile strength was about 1.0 MPa, and the tensile elongation at break exceeded 30%. The *M_n_* of PLLA nonwoven fabric was 122 kDa and the molar mass dispersity read 1.5. The DSC results showed lower *T_m_* and sharper melting peak for PLLA nonwoven fabric and the XRD spectrum exhibited a sharp peak and thus relatively high crystallization. The results indicated that the melt-spun PLLA fibers in the nonwoven fabric underwent rapid quenching and shear-induced orientation, yielding thinner yet uniform crystals with narrow thickness distribution (lower *T_m_*, sharper melting peak/XRD patterns).

Where three kinds of nonwoven fabrics (20, 40 and 80 g/m^2^) were compared, the thinnest nonwoven fabric was easily shredded during the suture process, and the thickest fabric obviously increased the overall volume of the occluder, which made it difficult for the occluder to be compressed into the delivery sheath. Therefore, the nonwoven fabric of 40 g/m^2^ was optimal and met the requirements as the flow-blocking membrane of an occluder.

### Fabrication of the PLLA occluder and evaluation of its physical performances

**Figure 3.**
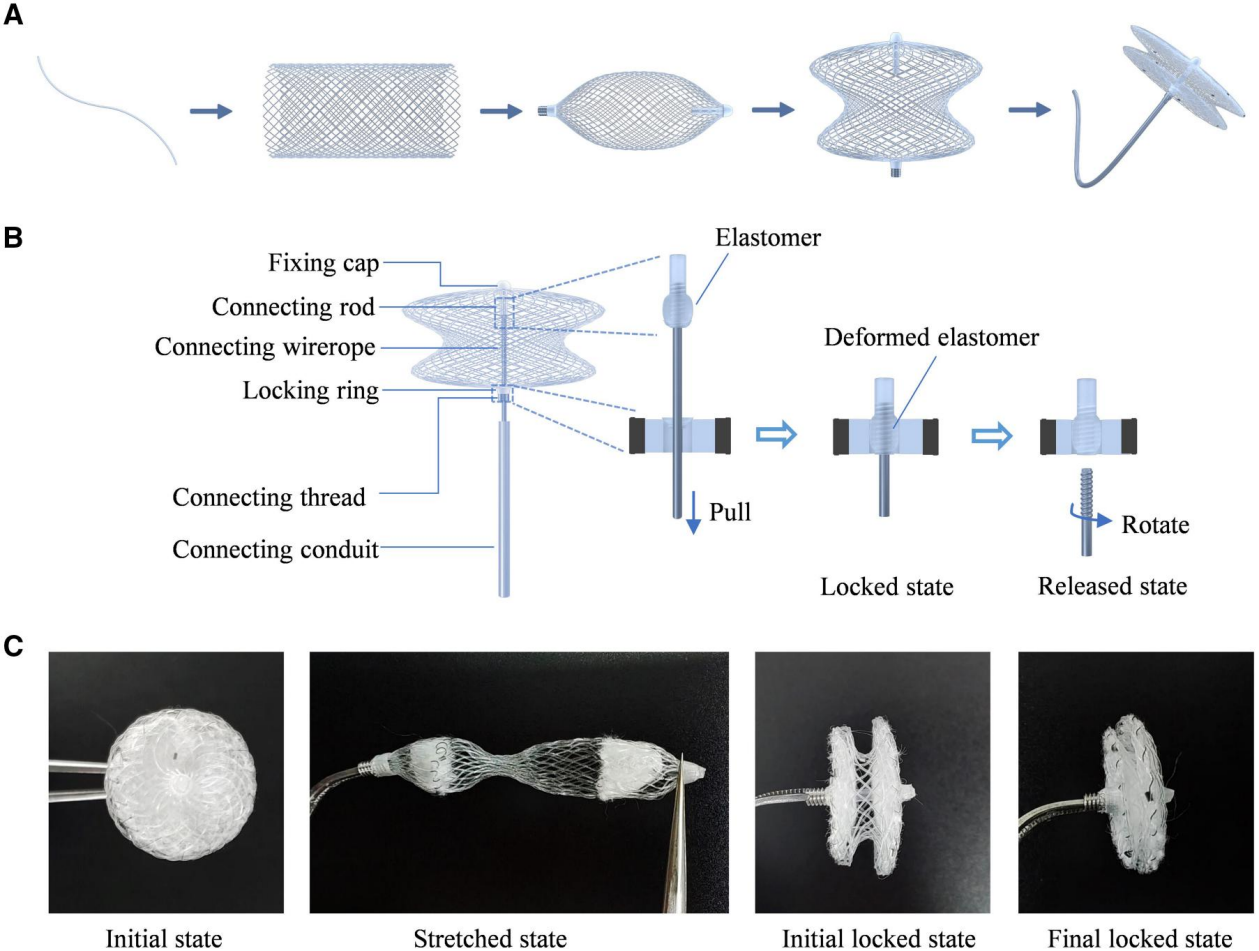
The structure design of the biodegradable PLLA occluder. (**A**) Schematic diagram of the fabrication process of the biodegradable ASD occluder. (**B**) Schematic diagram of the structure of the PLLA occluder and the locking procedure. An elastomer material is attached to the surface of the PLLA rod, and the rod is connected to wire rope via a thread. After the rod is pulled into the locking ring, the two disks are locked via the interference fitting. (**C**) Global views of the resultant PLLA occluder, initial state (left), stretched state (middle left), the initial locked (middle right) and the completely locked (right).

The fabrication process of the biodegradable occluder is schematically presented in Figure 3A, from the initial PLLA monofilament to the final occluder. We introduced some key components assembled to realize the controllable locking-unlocking function (Figure 3B). When the connecting wire rope is pulled through the locking ring, the elastomer can be deformed and achieve interference fit with the locking ring, and the left and right disks are locked together. Furthermore, the locked disks can be unlocked by pushing the connecting wire rope through the locking ring. When the occluder is positioned accurately, it can be released by rotating the connecting wire rope from the connecting rod. Figure 3C shows global views of the resultant biodegradable occluder in the initial state, stretched state, initial locked state and completely locked state. The real occluder is consistent with the designed structure and size, and no obvious damages on the monofilament and flow-blocking membrane were observed.

**Figure 4.**
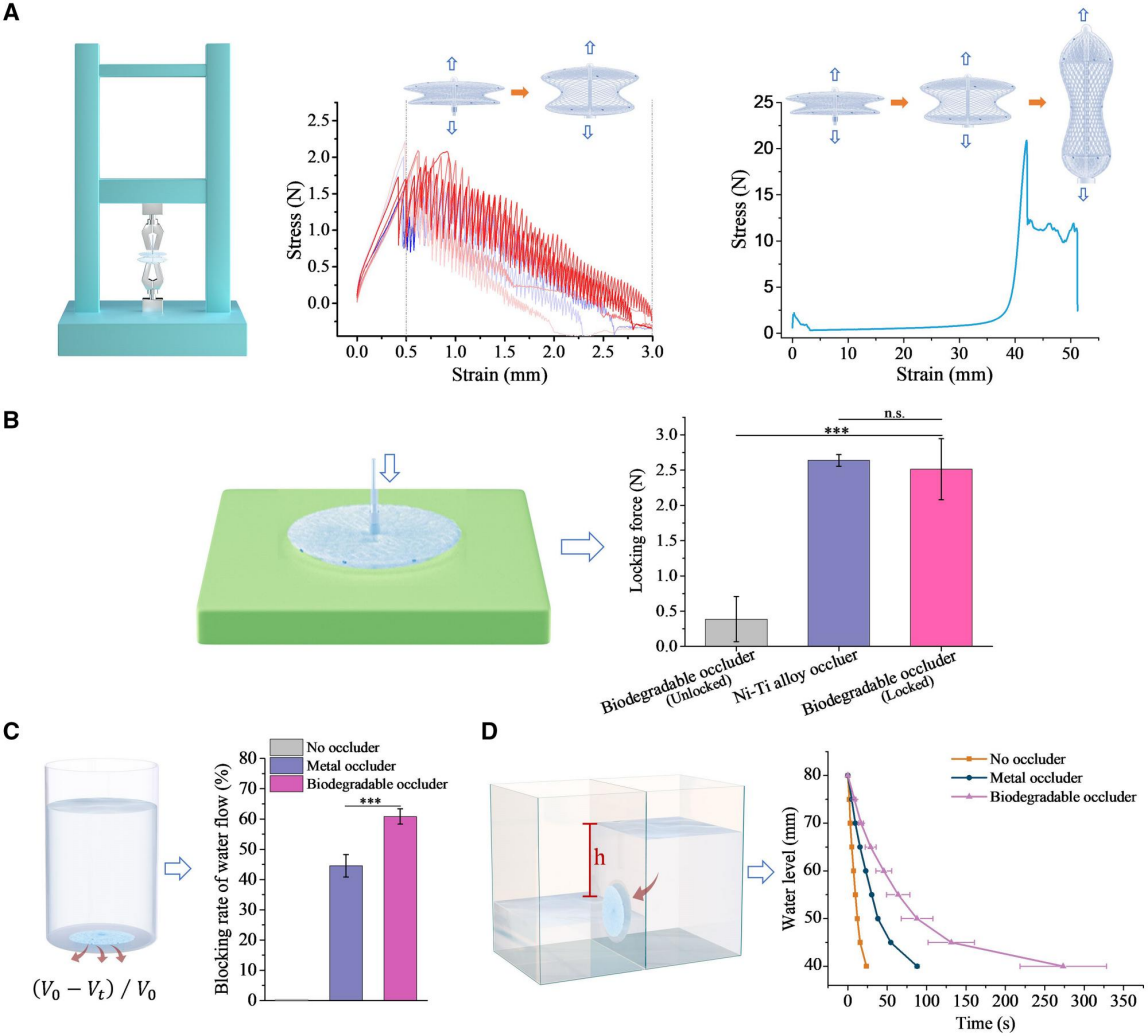
Methods developed by the authors to assess the performances of biodegradable PLLA occluders *in vitro*. (**A**) Schematic diagram of the tensile test of an ASD occluder. Typical stress–strain curves of the PLLA occluder from the locked state to the unlocked state, and a stress–strain curve of the PLLA occluder from the initial state of double disks to the tube-like delivery state. (**B**) Schematic diagram of the test of fixation force of an occluder, and the resultant fixation forces of metal and polymer occluders. (**C** and **D**) Schematic diagrams of the tests of the water-blocking effect with two different methods indicated, and the blocking effects of water flow for metal and polymer occluders.

The tensile performance, fixing firmness and water-blocking effect were measured via self-built methods to further evaluate the efficacy of a superelasticity-free occluder. Figure 4A shows the tensile stress–strain curves of the biodegradable PLLA occluder. The tensile stress that appears in the initial stage first rose and then decreased, indicating that the occluder was stretched from the locked state to the unlocked state, and the unlocking force was about 2 N, equivalent to the resilience force of the metal occluder. Afterwards, it was gradually stretched from the double-disk state to the final braiding-tubing state, and the tensile force of the occluder being stretched to the ultimate elongation state could reach 20 N. The fixation force of the biodegradable PLLA occluder kept a similar level to that of the Ni–Ti alloy occluder (Figure 4B).

The connection robustness between different components directly determines the use reliability of a biodegradable PLLA occluder. We employed tensile tests to measure the fixation forces between those components. According to Figure S2, the fixation force between the connecting rod and the connecting wire rope was 20 N, and those between the connecting rod and the braided frame, the locking ring and the connecting conduit, and the locking ring and the braided frame were about 5, 60, and 80 N, respectively. Among them, the lowest fixation force can reach 5 N (Figure S2), <2 N, the unlocking force of the occluder (Figure 4A). Therefore, the normal implant procedures might not cause damage to the components of occluder.

We also suggested a simple device to assess the water-blocking effect of an occluder to block a bottom hole under the pressure of water (Figure 4C). The results indicated that the blocking rate of water flow of the biodegradable PLLA occluder was significantly higher than that of the metal occluder. Figure 4D shows the changes in waterhead on both sides of the hole blocked by an occluder. It took the longest time for the waterhead to reach equilibrium after placing the biodegradable PLLA occluder, indicating that the polymer occluder exhibited the best water-blocking effect. The above results preliminarily confirmed the efficacy of this biodegradable PLLA occluder.

### *In vitro* degradation of the biodegradable occluder

**Figure 5.**
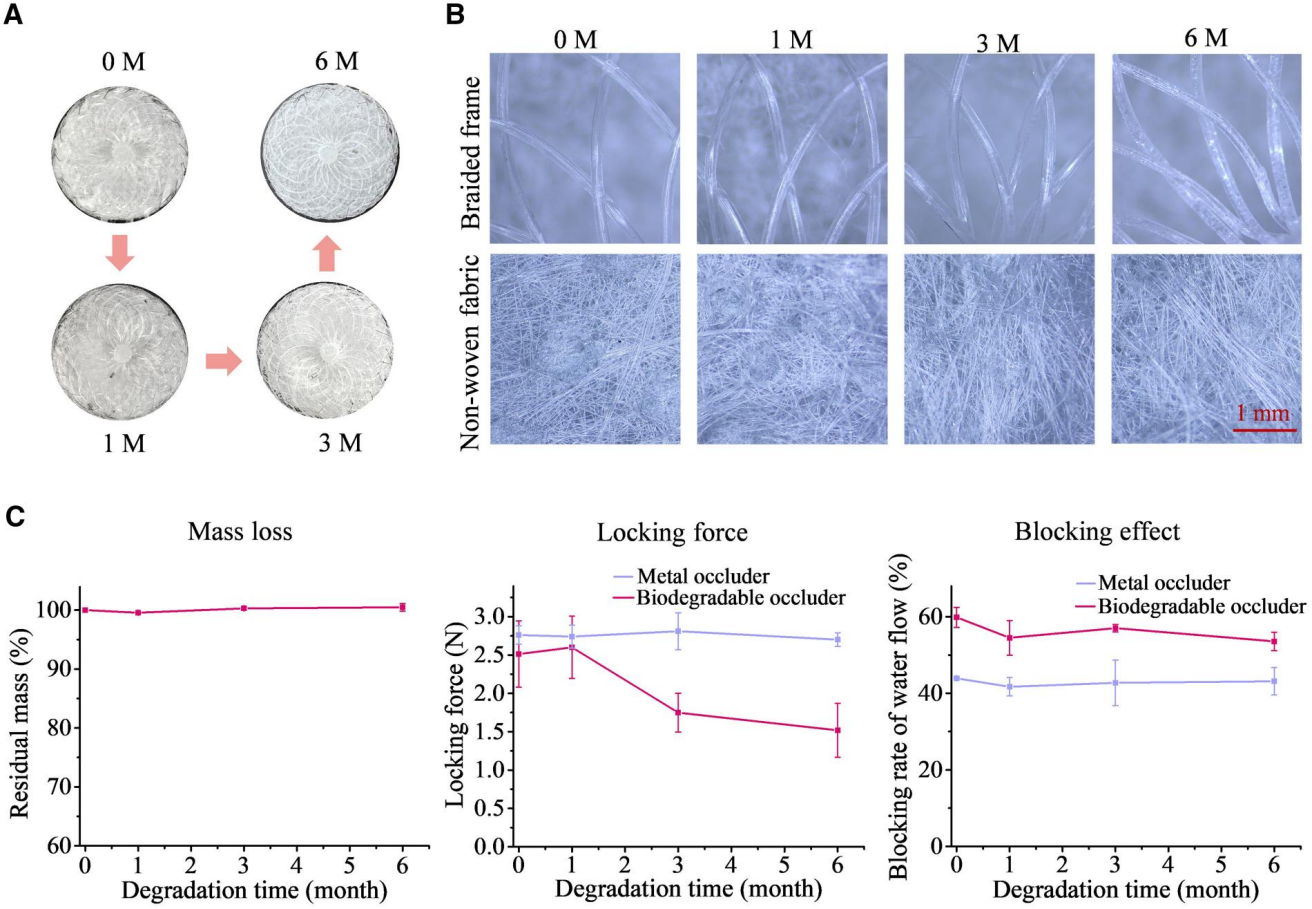
*In vitro* degradation. (**A**) Optical photographs of a PLLA occluder during 6-month degradation under PBS at 37°C. (**B**) SEM images of the frame and fabric parts of the occluder at the indicated time points. (**C**) Time dependence of residual mass, locking force and blocking rate of water flow of a PLLA occluder during degradation compared with a metal occluder (ASD occluder, KW081620, Dongguan Kewei Medical Instrument Co., Ltd).

Degradation in PBS can effectively simulate the *in vivo* degradation trend. The results from accelerated degradation at 60°C are shown in Figure S3, and those at body temperature are in Figure 5. The biodegradable PLLA occluder remained intact during 6 months of *in vitro* degradation at 37°C (Figure 5A). The PLLA monofilament was not broken and the nonwoven fabric was not damaged (Figure 5B). Localized whiteness on the monofilament was observed, which might be caused by the formation of microcrystalline areas during degradation. The atrial septal occluder generally completes endothelialization within 3–6 months after being implanted, and therefore the biodegradable occluder is required to maintain structural integrity, secure locking and stable water-blocking effect during this period. The 6-month degradation follow-ups indicated that no obvious mass loss occurred, and the locking force had remained at a high level even though there was a slight downward trend. Moreover, the water-blocking effect of the occluder had not significantly changed, and the blocking rate of water flow remained above the level of that the metal occluder kept (Figure 5C).

### *Ex vivo* simulated implantation

**Figure 6.**
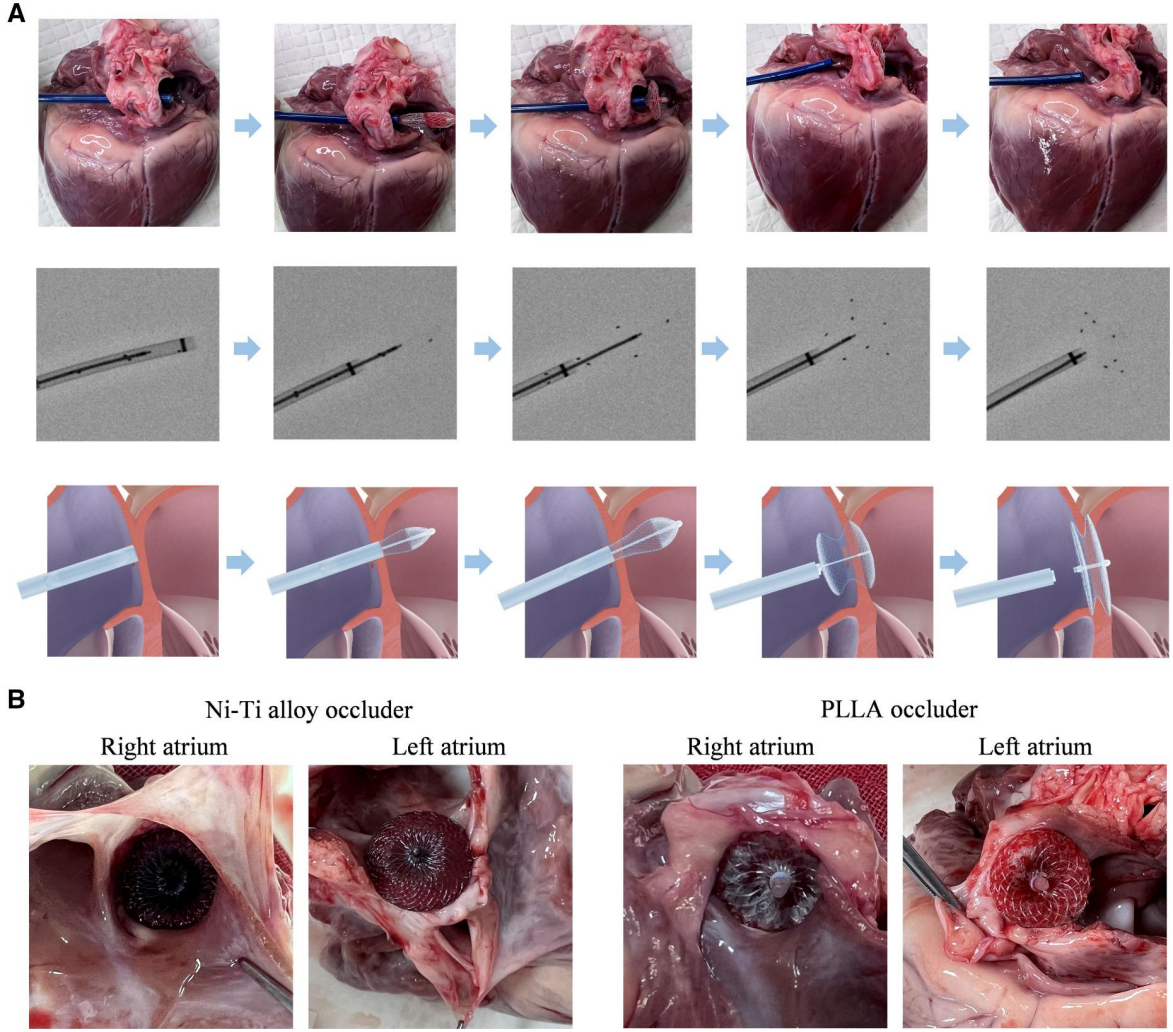
*Ex vivo* experiments to model the transcatheter delivery in a pig heart. (**A**) Global views of the operation stages (top), the radiographic photographs under DSA (middle), and the corresponding schematic presentations of transcatheter delivery of a PLLA device to occlude ASD (bottom). (**B**) Optical photographs of a metal ASD occluder (KW081620, Dongguan Kewei Medical Instrument Co., Ltd) and our polymer occluder in the porcine atrial septum.

We carried out *ex vivo* simulated implantation in the porcine heart model to evaluate the operability of the delivery, defect closure, locking and release of the new occluder. The biodegradable occluder was easily delivered to the target site, locked and released through a 10 F delivery sheath (Figure 6A, top). Since the polymer PLLA cannot be visualized under DSA, the status of the biodegradable occluder can only be determined by the position of the developing markers on the outline. When the biodegradable occluder was pushed out of the sheath, the position of the developing points was observed under DSA (Figure 6A, middle). The experiments verified the expected status of the occluder during implantation, including the occluder being compressed in the sheath, the left disk being pushed out, the waist being pushed out, the left disk being unfolded and the right disk being pushed out at the same time, and the double disks being locked. After being released, the occluder was firmly fixed to the atrial septum and well adhered to the wall, indicating a comparable implantation effect to that of the metal occluder (Figure 6B).

### Cell adhesion and subcutaneous implantation in rabbit

Considering that the fiber arrangement density of nonwoven fabrics may affect the adhesion and proliferation of endothelial cells, we examined cells on the surfaces of PLLA nonwoven fabrics at three different surface densities (20, 40 and 80 g/m^2^). As shown in Figure S4, the smaller the surface density of the nonwoven fabric was, the more loosely the PLLA fibers arranged, and larger fiber pores appeared.

**Figure 7.**
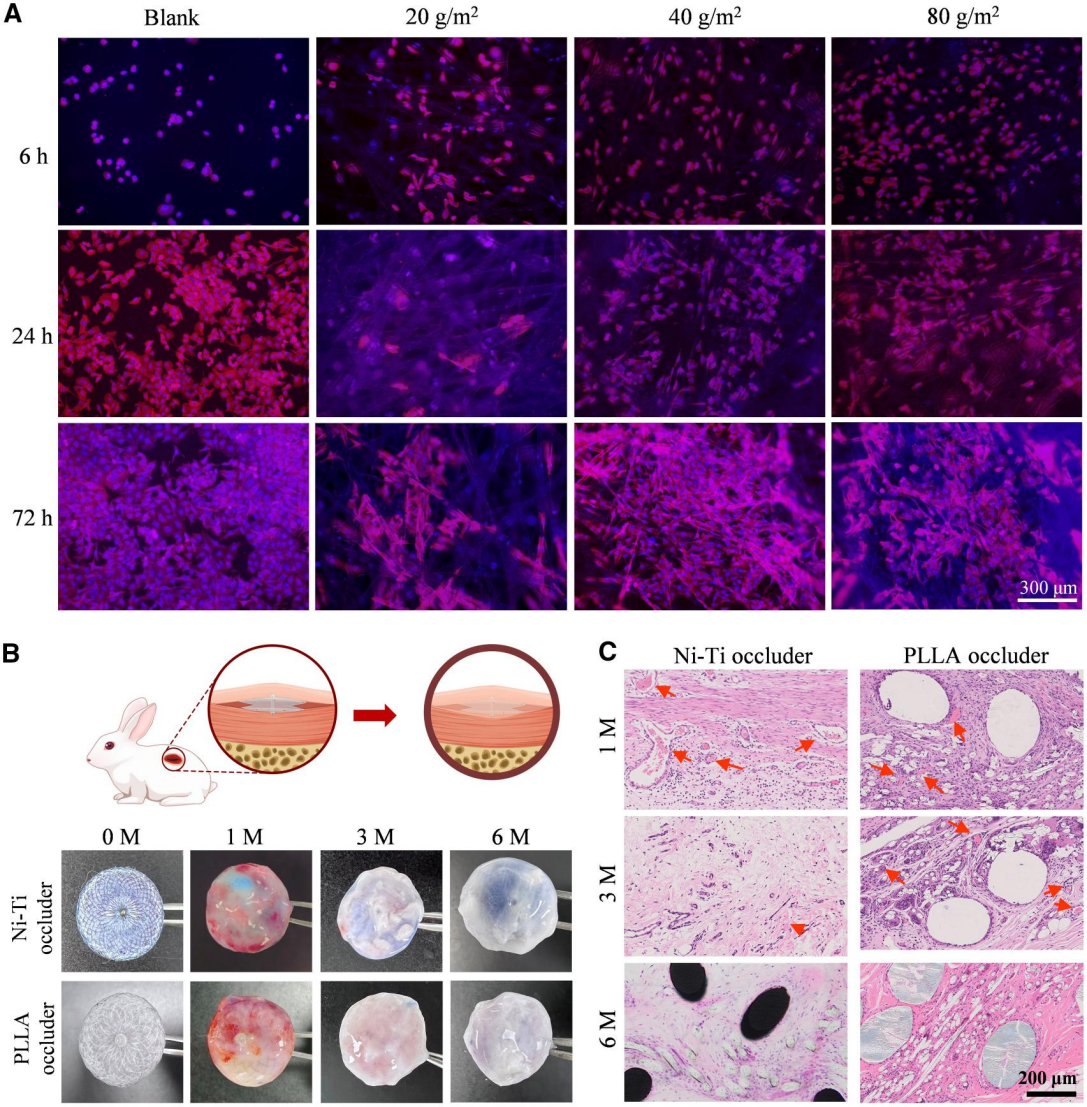
*In vitro* and *in vivo* examinations of cellular responses of the PLLA occluder. (**A**) Fluorescence images of human umbilical vein endothelial cells on nonwoven PLLA fabrics with the indicated surface densities. (**B**) Schematic diagram of a PLLA occluder implanted into the subcutaneous tissue of a rabbit, and optical photographs of metal and polymer occluders which were taken out after implantation in rabbit subcutaneously for the indicated time points. The raw Ni–Ti alloy filaments appear blue, and some blue area was observable, indicating the incomplete or thin endothelium on the occluder surface. (**C**) Optical micrographs of the pathological sections of HE-stained occluders. The arrows indicate the inflammatory cell aggregation. The black holes indicate the Ni–Ti alloy filaments. Only the group of the Ni–Ti alloy occluder implanted 6 months had applied the technique of hard-tissue slicing, and the black areas were observed in this metal group. The large white holes indicate the PLLA monofilaments, and the small white holes indicate the PLLA melt-blown fibers of membrane.

After 24 h of co-culture with endothelial cells inoculated on the surface of sterilized nonwoven fabrics, the results indicated that endothelial cells had entered the pores of the nonwoven fabric with the smallest surface density (20 g/m^2^) (Figure 7A). For the two nonwoven fabrics with larger surface densities, more endothelial cells adhered on the surface after 24 h of co-culture, and the cell number increased after 72 h of co-culture. Considering the cell adhesion effect, suturing effect and deformation softness, the 40 g/m^2^ nonwoven fabric was optimal as the flow-blocking membrane of the biodegradable occluder.

We also evaluated *in vitro* biocompatibility of our polymeric occluder using a standard approach relevant to cell adhesion and viability. No significant *in vitro* cytotoxicity was observed and the hemolysis was below 5% for the biodegradable PLLA occluder (Figure S5), which preliminarily confirmed that this occluder might meet the requirements of biocompatibility in the cardiovascular environment.

The *in vivo* biocompatibility was further examined after subcutaneous implantation of the biodegradable PLLA occluder into the back of a rabbit. Both the Ni–Ti alloy occluder and the biodegradable PLLA occluder were completely wrapped by fibrous tissue after one month of implantation. HE staining revealed inflammatory cells around the implants (Figure 7C). The inflammatory cell aggregation was significantly reduced or disappeared both for the Ni–Ti alloy and PLLA occluder after 3 months of subcutaneous implantation. The inflammatory cells almost disappeared at 6 months of subcutaneous implantation. In the intended application of cardiac occlusion device, rapid endothelial encapsulation (typically within 3–6 months) would isolate the occluder from surrounding tissues, which might mitigate inflammatory interactions. The observed low-grade inflammation in subcutaneous tissue aligns with biocompatibility standards for bioresorbable devices.

Moreover, the biodegradable PLLA occluder did not degrade significantly at 6 months of subcutaneous implantation, and the flow-blocking membrane (small white dots) and the monofilament (big white dots) did not deform or break significantly (Figure S6). Hence, the biodegradable PLLA occluder can maintain a relatively complete structural shape in the early stage of implantation, and facilitate complete endothelialization.

### *In vivo* transcatheter implantation and follow-ups in large animal experiments

**Figure 8.**
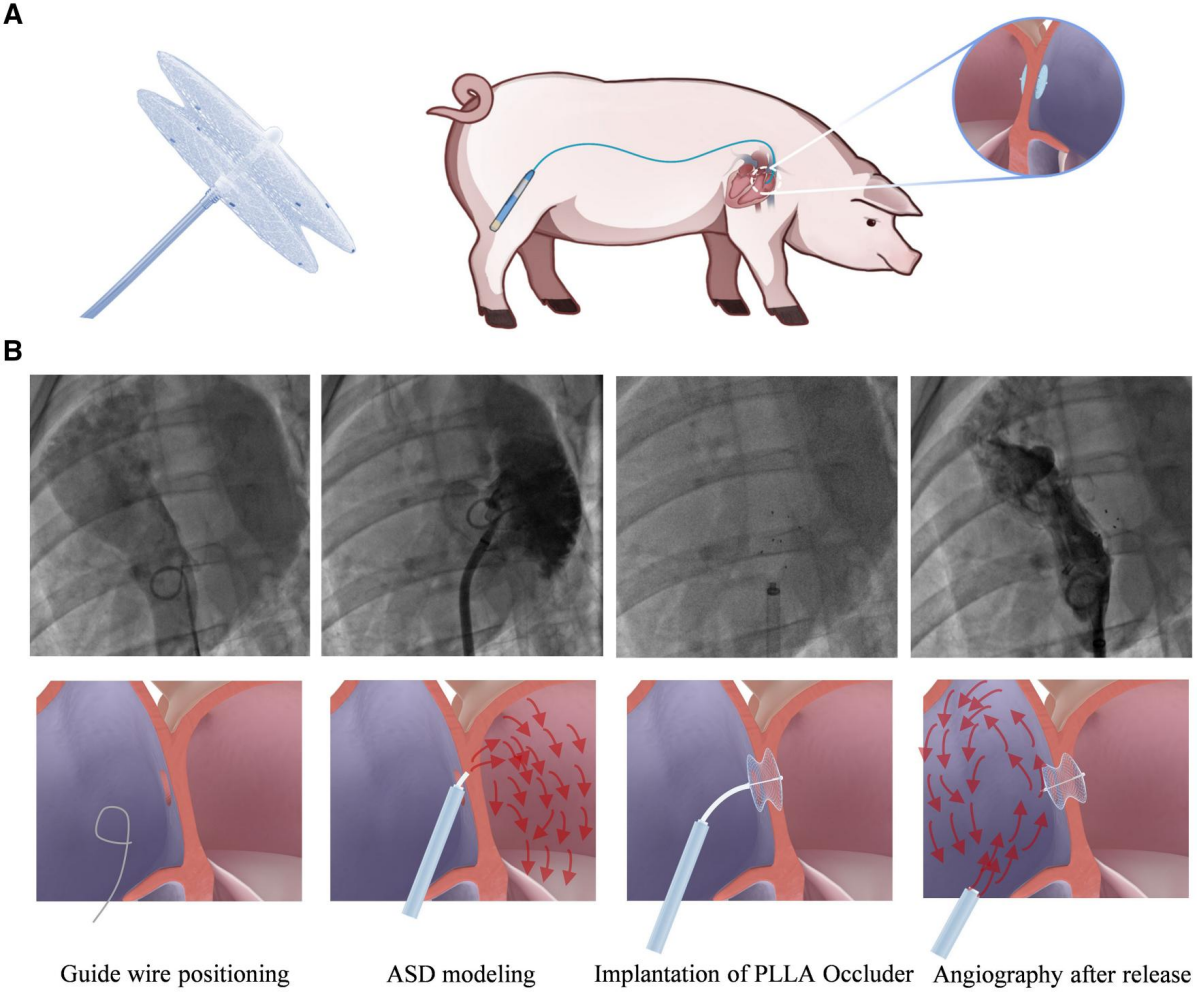
Mini-invasive implantation of our polymeric ASD occluder in a porcine model. (**A**) Schematic diagram of the biodegradable ASD occluder made of PLLA and its implantation in a porcine model. (**B**) X-ray images and corresponding diagrams of the atrial septum of a pig with guide wire positioning, ASD modeling, implantation of PLLA occluder and atrial angiography after occluder release.

The ASD occluder is generally delivered and implanted through the inferior vena cava to realize minimally invasive interventional treatment in clinical practice. Figure 8A shows a schematic diagram of the implantation of our biodegradable PLLA occluder into a pig heart through the femoral vein. First, the defect model of the atrial septum was built via atrial septal puncture, and the biodegradable PLLA occluder was successfully delivered to the target site via a 10 F delivery sheath. By externally controlling the connecting wire rope and connecting conduit, the double disks were fully deployed and locked together, and the connecting wire rope and connecting conduit were withdrawn in sequence, and the occluder was successfully implanted. The angiography results showed that the double disks were not completely attached to the atrial septal wall after the delivery sheath was completely withdrawn, but there was no residual shunt, and the defect site had still been effectively blocked (Figure 8B). Animal experiments preliminarily proved the safety and implantation feasibility of this biodegradable PLLA occluder with controllable locking structure in animals.

We would like to explain why cell adhesion and subcutaneous implantation were examined in rabbit but *in vivo* transcatheter implantation in pig. Rabbits have loose subcutaneous connective tissue, convenient for implanting biomaterials and observing localized cell adhesion, immune responses, or biocompatibility of implants. Pigs have cardiovascular systems anatomically and hemodynamically similar to humans, making them optimal for transcatheter procedures where precise navigation through blood vessels is required. Their larger size accommodates human-scale medical devices and allows for realistic simulation of clinical scenarios.

## Discussion

The recent decade has witnessed much progress in biomaterials [44–51]. Cardiac septal occluder is undergoing revolutionary development from non-degradable to biodegradable era [35–39, 52]. While biodegradable materials are crucial for many bioresorbable and environment-friendly devices [53–61], the structure design and feasibility of use are more important for biodegradable occlusion devices. The present work combines the selection of materials and structural design to develop a biodegradable PLLA ASD occluder and distinguishes itself by using controllable locking structure, a suitable membrane for better endothelialization and performance evaluations.

### Controlled locking structure design for firm closure

The term superelasticity originates from shape memory alloys like nitinol, where the material can recover large deformations upon stress removal due to reversible martensitic-austenitic phase transformations. Sometimes, this term merely refers to the ability of significant elastic recovery. Superelasticity is critical for medical devices such as metal occluders, which require precise deployment and shape recovery. Considering the lack of superelasticity of most polymers, it is impossible for a biodegradable occluder composed of PLLA to spontaneously recover to its desired state and difficult for a physician to implant this occluder with firm closure. This has been indicated in the literature [30, 31, 37] and supplementally illustrated by our finite element simulations (Figure S7). We attempted to improve the structure design to compensate for the inherent lack of significant elasticity of PLLA. In this work, a controlled locking structure was conceived and applied in our biodegradable polymeric occluder (Figure 3). Unlike traditional superelastic nitinol occluders, this polymer occluder achieved the due post-implanted shape via a controlled locking structure and a serial of transcatheter operations, where the connecting wire rope was pulled through the locking ring, and the elastomer can be deformed and fit with the locking ring, achieving a controllable locking and unlocking. The developing markers were well installed on the outline of the occluder to clearly observe the position and status under DSA. This structural design effectively makes up for the problems of insufficient resilience in shape recovery and makes it possible to develop a biodegradable polymeric occluder.

### Moderate surface density of nonwoven fabric for better cell adhesion and proliferation

Cell-material interactions are important for tissue regeneration and implanted biomedical devices [62–70]. The double-disk biodegradable PLLA occluder was composed of a braided mesh and a flow-blocking membrane, and the flow-blocking membrane was sutured under the braided mesh. When endothelial cells contacted the outer surfaces of such an occluder, they adhered, proliferated and migrated on the flow-blocking membranes, and finally wrapped the braided mesh to form complete endothelial tissue. Therefore, the characteristics of the flow-blocking membrane might affect the behavior of endothelial cells, and a moderate surface density of nonwoven fabric is beneficial for rapid endothelialization on the occluder surfaces (Figure 7).

### Establishment of *in vitro* methods to evaluate the performance of an occluder for interventional treatment

Without relevant evaluation standards for a biodegradable occluder, it is difficult to effectively evaluate the performances, especially for fixation force of locking, water-blocking effect, degradation and performance change during degradation. Wang and his colleagues reported an effective evaluation method of fixation force for their biodegradable occluder [37]. Nevertheless, many other performance evaluation methods are rarely reported. In the present study, we developed a series of methods to access an occluder *in vitro* (Figure 4). While the fixation force was selected as the test index to evaluate the connection reliabilities between the joints of the components of occluder, we introduced more design to perform evaluation toward the interventional treatment. As for the locking reliability and fixing firmness of the occluder, the fixation force test was made with a model that simulates an atrial septal hole and with the metal occluder as a control. The water-blocking effect is a key indicator for the evaluation of residual shunt, and we proposed two methods to test the blocking rate of water flow with simulation models. Besides, degradation in PBS buffer can effectively simulate the *in vivo* degradation trend, and we examined both accelerated and simulated 37°C degradations to evaluate the performance changes of the biodegradable occluder (Figure 5). To evaluate whether our biodegradable occluder was easily delivered, locked and released through a 10 F delivery sheath, and whether the position of the developing points can be clearly observed under DSA, an *ex vivo* simulated implantation in a porcine heart model was carried out. These methods are helpful for the effective performance evaluation of an occluder.

### Safety and efficacy of the biodegradable PLLA occluder

The connection reliability between different components determines an important aspect of the safety of a biodegradable PLLA occluder. The fixation forces between the various components of our occluder are rather high, and normal implant procedures cannot cause damage to the components of occluder, which ensures its safety and reliability. The biodegradable PLLA occluder in the locked state maintained a locking force of about 2 N (Figure 4), equivalent to that of the metal occluder, ensuring that it can be firmly locked in ASD. According to the blocking rates of water flow, this biodegradable polymeric occluder exhibited a better water-blocking effect than that of the Ni–Ti alloy occluder, possibly owing to the more flexibility of a polymer than a metal during locking and sealing. The delivery, deployment, locking and unlocking operations were smoothly completed, showcasing the operability of the transcatheter implantation. *In vitro* simulated degradation indicated that the biodegradable occluder kept structural stability for at least 3 months, providing basic guarantee for early endothelialization on the surface. Moreover, the position and status of the biodegradable occluder were well determined through the developing markers under DSA. *Ex vivo* simulated implantation in the pig heart model performed well; the occluder was firmly fixed to the atrial septum and well adhered to the wall.

The biological safety of the biodegradable occluder was verified *in vitro* and *in vivo* (Figures 6 and 7). The chemistry pentathlon tests, cytotoxicity and hemolysis tests conducted in accordance with medical device regulations all met the standards. In the rabbit subcutaneous implantation test, few inflammatory cells aggregated in the tissues around the biodegradable PLLA occluder in the early stage, and no obvious inflammatory cell aggregation was observed 3 months after implantation. According to the *in vivo* interventional experiment, no abnormal acute complications occurred during and after the implantation.

The biodegradable PLLA occluder was successfully delivered to the target site through the inferior vena cava by interventional treatment (Figure 8). After being pushed out from the 10 F delivery sheath, the double disks of the occluder were fully unfolded and locked together to achieve ASD occlusion. After releasing the occluder, the delivery system was smoothly withdrawn. The angiography results showed no obvious residual shunt after the implantation of the occluder. Animal experiments have preliminarily proved the efficacy of this biodegradable PLLA occluder.

### Limitations

The main characteristics of our biodegradable occluder are summarized as follows: (i) the chief raw material is PLLA and can be completely degraded in ∼3 years, which potentially enables regeneration of the heart septum tissue; (ii) the structural design can realize controllable locking, and the developing markers installed on the outline of the occluder can help to observe the position and status under DSA, which effectively compensates the insufficient resilience, difficulty in recovery and visuality of a polymer occluder; (iii) the moderate surface density of nonwoven fabric enables a complete endothelial layer on the outer surfaces of the occluder, and meanwhile meets the requirements of membrane suture and deformability of the occluder.

This paper demonstrates the feasibility of the biodegradable polymeric occluder via a series of *in vitro* performance evaluations and animal experiments. Nevertheless, there are still some issues that should be improved toward clinical translation: (i) it is required to establish the full standards to guide product design and performance evaluations for biodegradable cardiac occluders, which depends upon further academic research and relies on cooperation among pertinent institutions, hospitals and companies; (ii) only the adhesion and proliferation of endothelial cells on nonwoven fabrics with different surface densities have been compared, and more material factors on cell behaviors are expected to be examined; (iii) although the biodegradable PLLA occluder can achieve the same fixation force and water-blocking effect as the metal occluder, a series of surgical operations are necessary to complete the delivery and device locking owning to lack of superelasticity and radiopacity, which increases the difficulty of training a doctor; (iv) in the large animal experiment, the ASD model was completed by the atrial septal puncture needle and sheath. Therefore, the present animal experiment cannot fully reflect the implantation effect of the biodegradable PLLA occluder in patients.

## Conclusions

A biodegradable polymeric occluder with a controllable locking structure has been designed and fabricated, and a series of self-built evaluation methods have been established. The *in vitro* physical performances and PLLA degradation, *ex vivo* simulated implantation, *in vivo* subcutaneous implantation, and interventional closure in a porcine model have confirmed the safety and efficacy. The limitations and perspectives are finally indicated. The present study sheds insight into the biodegradable medical device for the interventional treatment of congenital structural heart diseases.

## Supplementary Material

rbaf016_Supplementary_Data
