# Supplementary material for: Biodegradable polymeric occluder with controllable locking structure for closure of atrial septal defect via interventional treatment
Source: Regen Biomater. 2025 Mar 20;12:rbaf016. doi: 10.1093/rb/rbaf016 (PMC12005900; doi:10.1093/rb/rbaf016)
Supplement: rbaf016_Supplementary_Data [file rbaf016_supplementary_data.pdf]

Supplementary Materials

Biodegradable polymeric occluder with controllable locking structure for closure of atrial septal defect via interventional treatment

Daokun Shi[1], Yahong Kang[1,2,3], Weijie Wang[2,3], Ruili Liu[1], Quansheng Tang[2,3], Zhaomin Li[2,3], Hongyan Jiang[2,*] and Jiandong Ding[1,*]

[1]State Key Laboratory of Molecular Engineering of Polymers, Department of Macromolecular Science, Fudan University, Shanghai 200438, China

[2]Shanghai Key Laboratory of Interventional Medical Devices and Equipment, Shanghai MicroPort Medical Group Co., Ltd, Shanghai 201203, China

[3]AccuPath Group Co., Ltd, Jiaxing 314000, China

[*]Corresponding authors. E-mail: jdding1@fudan.edu.cn (JD Ding); hyjiang0241@outlook.com (HY Jiang)

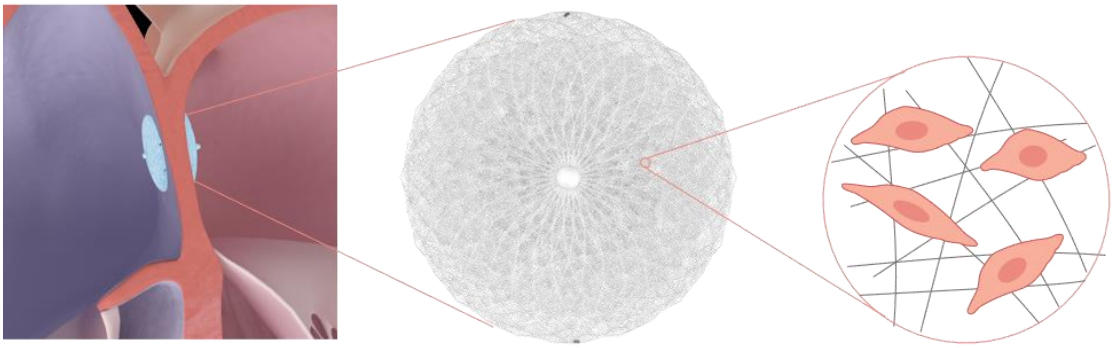

Figure S1. Schematic diagram of the status of an atrial septal defect (ASD) occluder after being implanted and the surface topography of the occluder.

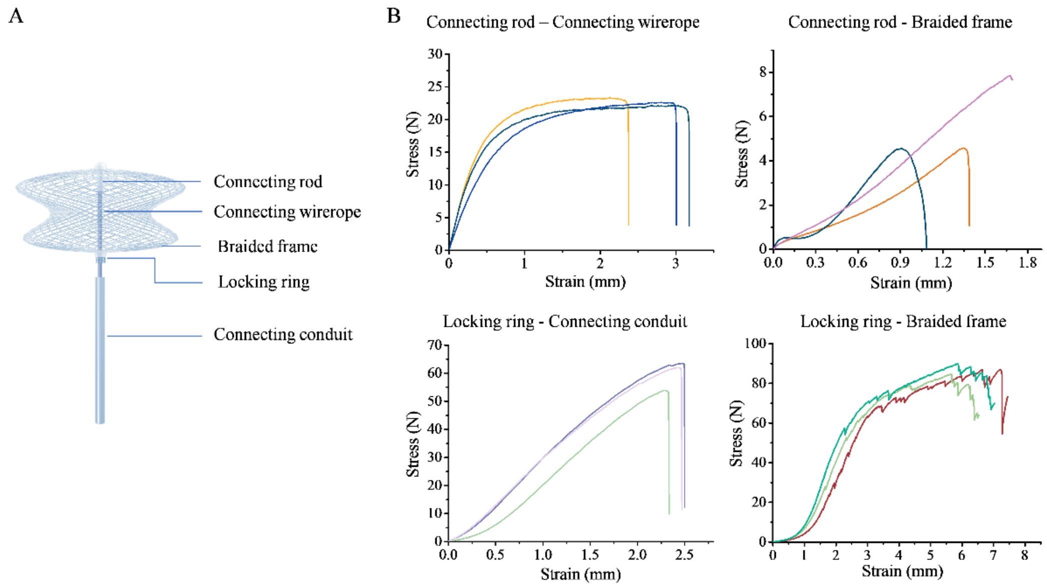

Figure S2. Mechanical tests of the main parts of the poly(L-lactide) (PLLA) occluder. (A) Schematic description of components of the PLLA occluder. (B) Stress-strain curves of the indicated different components of the PLLA ASD occluder.

The long-term degradation trend of the biodegradable occluder can be primarily mimicked in the short term by an accelerated degradation such as at temperature higher than the body temperature. Figure S3 shows the global views of the biodegradable PLLA occluder in phosphate buffer saline (PBS) at 60 °C. After 2 weeks of degradation, the monofilaments had broken, but the overall structure of occluder remained intact. After 4 weeks of degradation, the braided skeleton and the flow-blocking membrane were severely damaged, the occluder structure was deformed, and obvious mass loss began to occur. After 8 weeks of degradation, the biodegradable occluder was degraded into broken particles, and the mass loss reached about 50%.

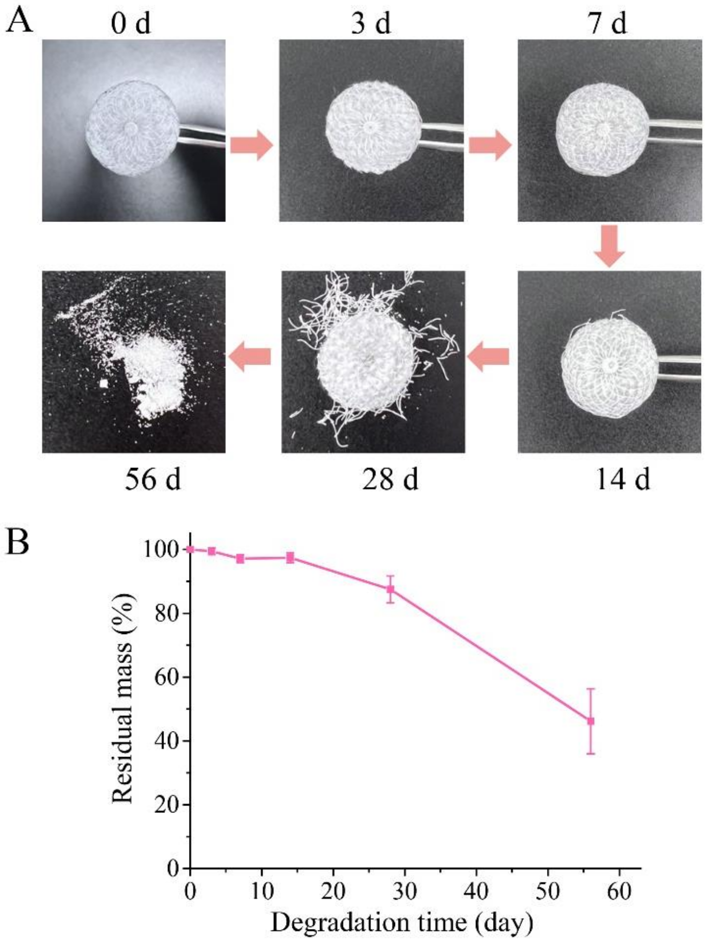

Figure S3. *In vitro* accelerated degradation in PBS at 60 °C. (A) Optical photographs of the PLLA occluder and (B) residual mass during 56 days of the accelerated degradation.

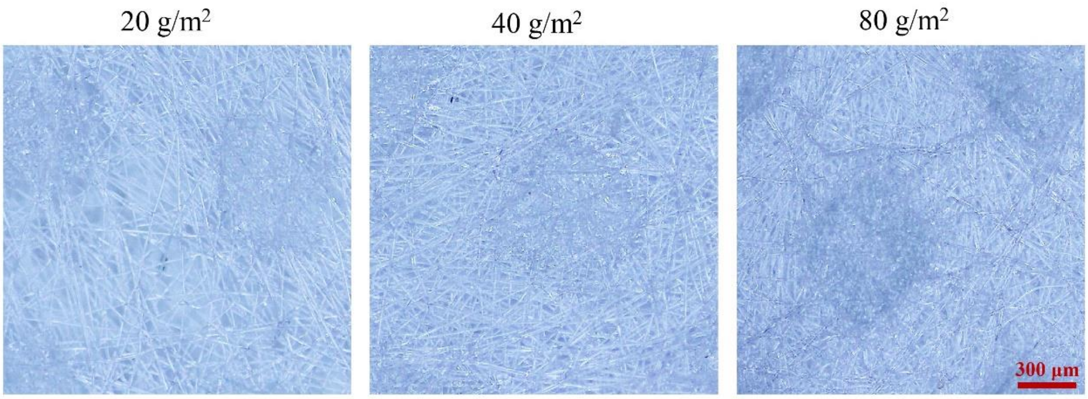

Figure S4. Optical photographs of nonwoven PLLA fabrics with the indicated surface densities. The fabric was itself in white, and the bluish colure came merely from the microscopic imaging.

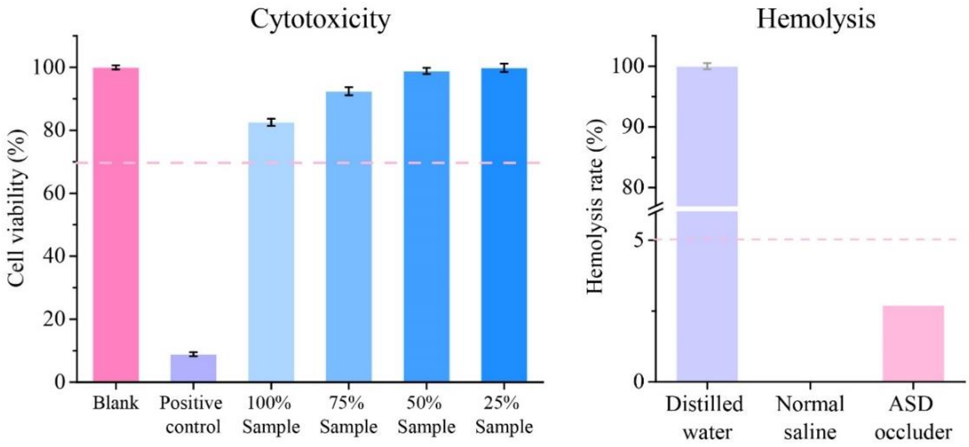

Figure S5. *In vitro* cytotoxicity and hemolysis of the PLLA occluder. The dashed lines indicate the acceptance criteria suggested by ISO.

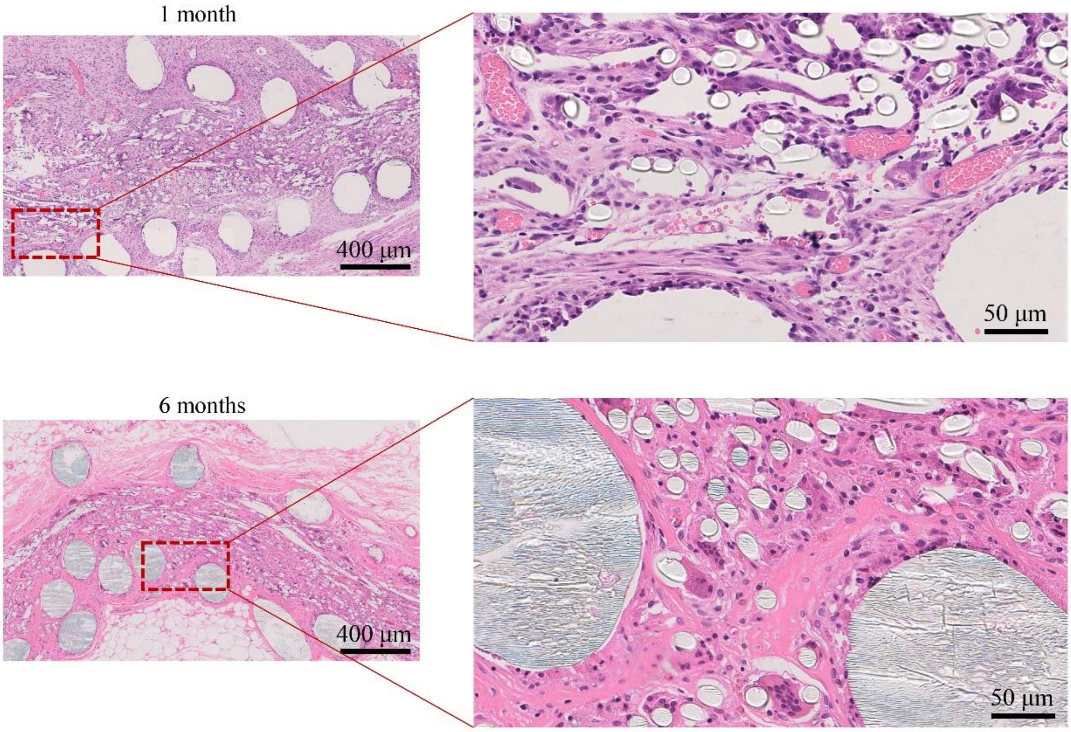

Figure S6. Optical micrographs of the pathological sections stained with hematoxylin-eosin (HE) of the implanted PLLA ASD occluder at the indicated post-implantation time points.

Through finite element simulation analysis, the skeleton of the biodegradable occluder without a locking device was stretched to the delivery state (tubular structure) by external force. When the external force was released, the occluder only partially recovered its shape. The result indicated that the polymeric occluder without superelasticity cannot realize self-recovery and stable fixation.

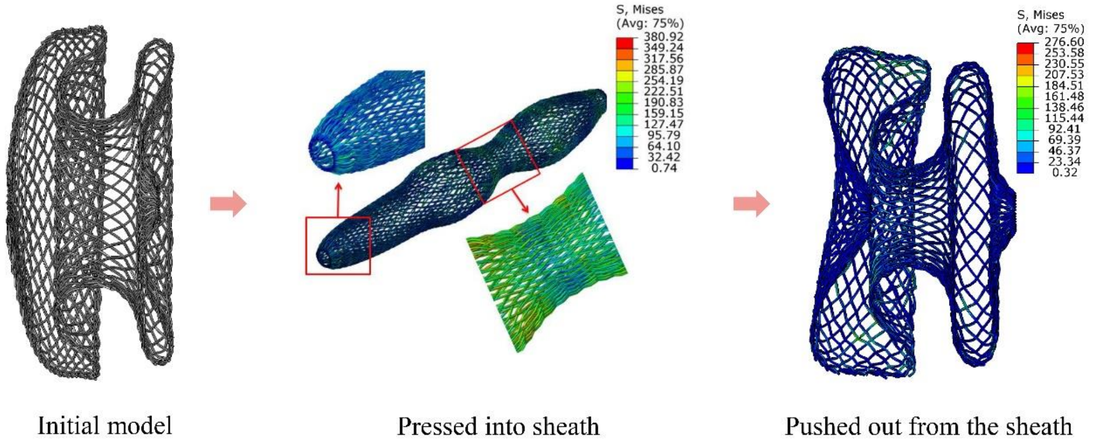

Figure S7. Finite element simulation to analyze the structure states of a PLLA occluder without the locking structure during transcatheter delivery and release. The results indicate that the elasticity of PLLA itself is insufficient to clamp the two disks after being released.
